# Experimental observation of flat focusing mirror based on photonic jet effect

**DOI:** 10.1038/s41598-020-65292-7

**Published:** 2020-05-21

**Authors:** Igor V. Minin, Cheng-Yang Liu, Yu-Chih Yang, Kestutis Staliunas, Oleg V. Minin

**Affiliations:** 1 Tomsk State Politechnical University, Tomsk, 36 Lenin Avenue, 634050 Russia; 2 Tomsk State University, Tomsk, 30 Lenin Avenue, 634050 Russia; 3 Department of Biomedical Engineering, National Yang-Ming University, 11221 Taipei City, Taiwan; 4 ICREA, Passeig Lluís Companys 23, 08010 Barcelona, Spain; 5 UPC, Dep. de Fisica, Rambla Sant Nebridi 22, 08222, Terrassa, Barcelona, Spain

**Keywords:** Micro-optics, Sub-wavelength optics

## Abstract

In this work, we experimentally demonstrate that a thin rectangle dielectric-metal structure can have a function of a flat focusing mirror based on photonic jet effect in reflection mode. Using polydimethylsiloxane (PDMS) rectangle with size length of 10 μm and wavelength-scale thickness of 1 μm on the top of a silicon wafer, we have built a flat mirror which focuses an incident beam at the focal length changing from 1.38 μm to 11.67 μm upon tuning the beam incidence angle from 30° to 75°. The focusing properties of such a mirror persist in the wavelength range of 405 nm to 671 nm. Our approach can be extended to realize other optical functionalities by properly controlling rectangle dimensions and materials. This flat focusing mirror is able to guide the incident beam in free space without perceptible diffraction at the distance equal to the photonic jet length and suitable for small-scale photonic circuits.

## Introduction

Light focusing mirrors have been keen to academic interest since ancient times. Even Plutarchos (201–120 BC) describes huge focusing mirrors and mirrors to set fire to Roman ships attacking city of Syracuse in 212 BC, designed and built by Archimedes^1^. The well-known principle of focusing light by classic focusing mirrors is due to their curved surfaces. Recently, researchers have proposed more sophisticated architectures of focusing mirrors, such as near-field flat focusing mirrors (FFM), which have interesting applications in optics and photonics. In the first instance, the sub-wavelength flat dielectric high-contrast gratings may act as a reflectors with focusing functionalities^2–4^. In the second instance, the broadband FFM can be designed in metal-insulator-metal configuration^5^. Artificial planar configurations of FFM, capable to focus the narrow beams, are considered as well: Bragg-like mirrors, consisting of specially designed dielectric multilayers^6^, and periodically modulated surfaces on a subwavelength scale^7^. A review of near-field FFMs with different configurations of photonic crystals is given in the reference^8^. It could be noted that a flat focusing lens based on a flat chirped dielectric mirror cannot focus a radiation at normal incidence^9^.

Another approach to building a FFM is based on the photonic jet (PJ) effect^10–13^ in reflection mode. In this case, the dielectric cuboid is illuminated with a plane wave and a PJ (the beam focus) appears close to the cuboid surface from the opposite direction of the incident electromagnetic field^14,15^. The capabilities of localized electromagnetic field (near-field focusing) using mesoscale 3D dielectric cuboid placed on flat surface and working in reflection mode are studied under plane-wave illumination. The focusing properties from the dielectric cuboid have been evaluated at the fundamental and first frequency harmonics under normal and oblique incidence. The similar results are discussed for hemi-spherical dielectric particle placed on flat metal surface^16^ and for a near-unity-refractive-index sphere on a dielectric substrate with high index contrast^17^. Based on PJs formed in the reflection regime, new modifications of a subwavelength, standing-wave optical trap are proposed to stabilize the position of a particle at a certain distance from the surface^18^. It could be noted that such design of focusing mirrors and the configuration of PJ formation in reflection mode are principally different from those in conventional far-field lens or thin lens based on engineered metasurface thin lens^19^ and subwavelength gratings.

However, the experimental investigations of PJ formations and their applications have been developed using dielectric particles only in the transmission regime (along the propagation direction of the incident radiation). In this study, we experimentally demonstrate a flat focusing mirror in optical frequency without predefined optical axis. The specifically designed mesoscale rectangle shaped structure from conventional dielectric is placed on a high refractive index plate. We refer such structure as a thin rectangle (TR). The multi-wavelength response is evaluated for sub-wavelength focusing in the reflection regime. The PJ performance is directly observed from dielectric TR under oblique incidence.

## Results and Discussion

First, the capability to produce PJ in reflection mode is numerically studied using dielectric TR illuminated by laser beam under different oblique angles. Since the beam width of 1 mm is much broader than dielectric structure size (10 μm), the incident laser beam is considered to have a quasi-plane wavefront. Moreover, the beam divergence of these lasers is less than 1.5 mrad and the light source is almost collimated beam in short propagation distance (<50 cm). The focusing properties of the TR are evaluated in terms of focal length and full width of the reflected beam at half-maximum (FWHM)^20^ depending on wavelength. The simulation results demonstrate that both the length and the width of the focal voxel decrease with the increase in the illumination angle. The field intensity in the focus decrease as well, and the incident angle is approximately equal to the reflection angle. Finally, the designed structure has been fabricated and characterized at three wavelengths of 405 nm, 532 nm, and 671 nm under oblique incidence by rotating the light source to vary the incident angle from 30° to 75°.

**Figure 1:**
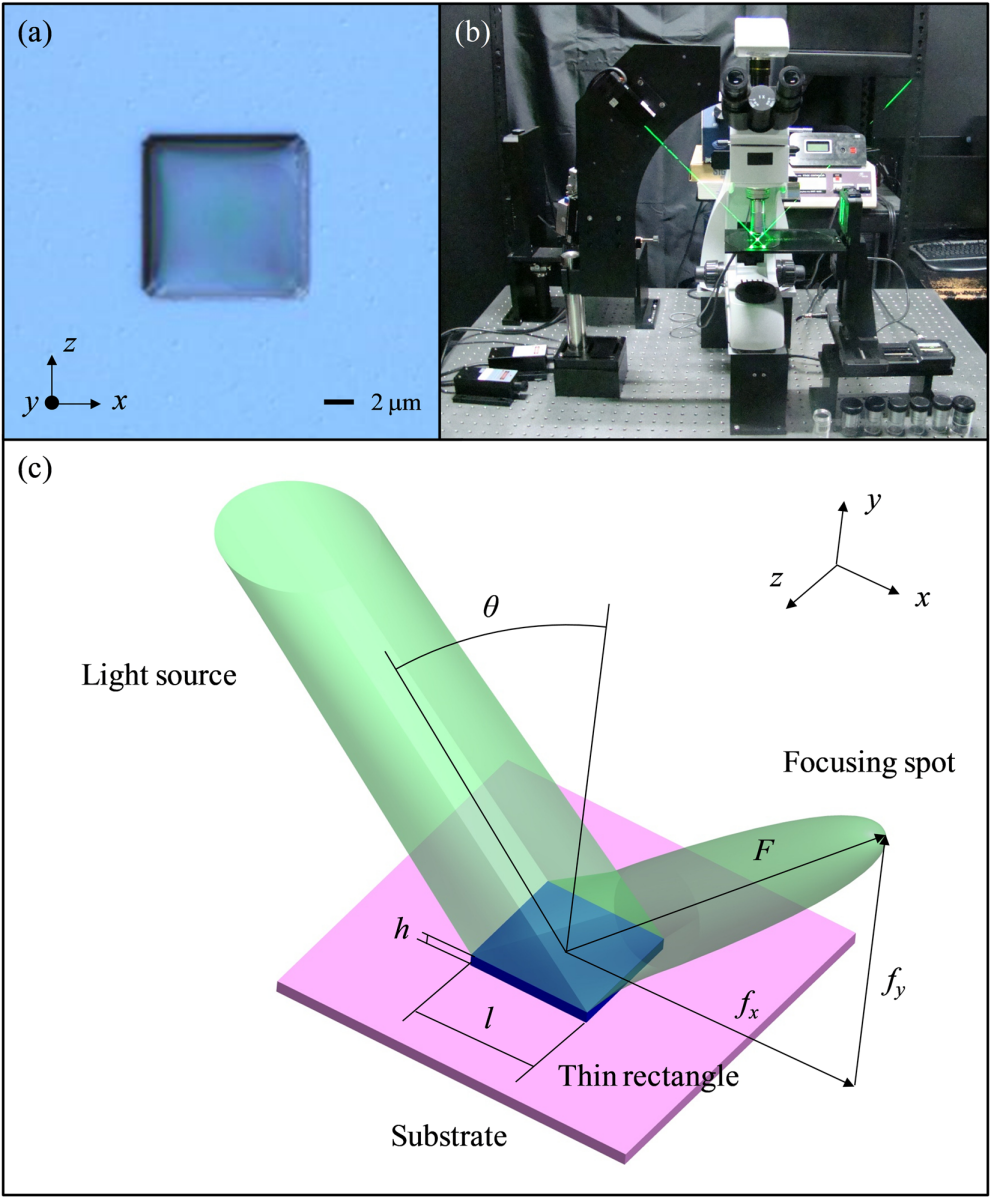
(**a**) Microphotograph of a single dielectric thin rectangle on a substrate. (**b**) Photograph of the scanning microscope system with an oblique illumination. (**c**) Schematic diagram of the proposed flat dielectric thin rectangle working in reflection mode. Microsoft PowerPoint 2016 is used to draw (**c**).

In this report, the 3D dielectric object in the form of flat TR working in the reflection regime is schematically shown in Fig. 1(c). The dielectric material of the TR is polydimethylsiloxane (PDMS). This TR has lateral dimensions *l* = 10 μm (*d* = 24.69λ_0_ along *x* and *z* axes) and thickness *h* = 1 μm (*h* = 2.47λ_0_ along *y* axis), and is placed on the center of the 4 inches (101 mm) silicon wafer with a small absorption (refractive index *n* = 5.567 + 0.386i), as shown in Fig. 1(a). The normalized fundamental frequency corresponds to the wavelength λ_0_ = 405 nm. The refractive indices of PDMS at the wavelengths of 405 nm, 532 nm, and 671 nm are 1.42, 1.41, and 1.409, respectively^21^. The refractive index contrast between the TR and supporting plate is about 3.9. To evaluate the PJs produced in reflection mode, the whole structure is located in air (*n* = 1).

**Figure 2:**
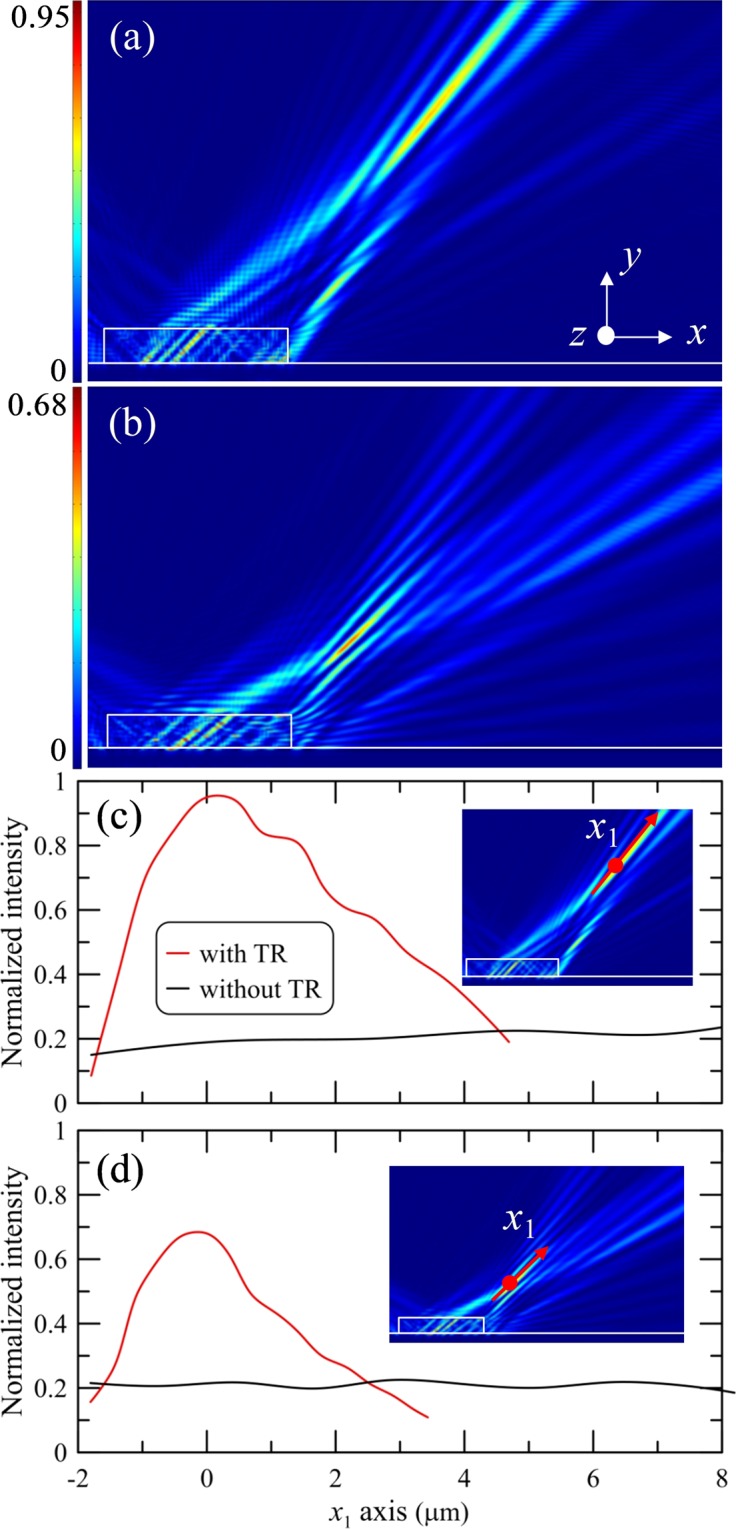
Simulation results of normalized power flow patterns for the flat dielectric thin rectangle in reflection mode at incident angles (**a**) 45° and (**b**) 60°. The incident wavelength for the thin rectangle is 405 nm. Normalized power distributions along propagation direction with and without thin rectangle at incident angles (**c**) 45° and (**d**) 60°. The inserts in (**c,d**) indicate the location (red line) of the power distributions along propagation direction (*x*_1_ axis in insert). The origin (red point) of *x*_1_ axis is located at the point of maximum intensity.

The focusing performance of the PJs is evaluated at the normalized fundamental frequency using 3D dielectric TR by varying the illuminating angle. For instance, the numerical results of the power flow on the *xy*-plane at incident angles of 45° and 60° are shown in the Fig. 2(a,b). The analysis shows that the focal lengths of the PJs are *f*_*y*_ = 15.65 μm (38.6λ_0_) at 45° and *f*_*y*_ = 11.6 μm (28.6λ_0_) at 60°, which corresponds to the conditions of a far-field zone (*f*_*y*_ ~ 30λ_0_). This is an expected result due to the fact that the lateral dimensions of the TR are 24.69λ_0_ which is significantly more than a few wavelengths^14^. In Fig. 2, the multiple reflections within the TR are also clearly visible because of the flat shape of the dielectric structure. The reflection angle is approximately equal to the angle of incident radiation. Figure 2(c,d) show the normalized power distributions along propagation direction with and without the TR at incident angles 45° and 60°. The field intensity profile along PJ is determined as follows. We first find the point of maximum intensity of PJ. The longitudinal profiles in Fig. 2(c,d) are acquired as a section of the intensity distribution along the straight line parallel to propagation direction *x*_1_ and passing through the point of maximum intensity. The origin of *x*_1_ axis is located at the point of maximum intensity. With an increase in the incident angles from 45° to 60°, the maximal field intensity at the localization region (PJ) decreases about 28% (from 0.95 to 0.68 in relative unit) and the PJ length decreases from 1.12 μm to 0.92 μm. A comparison of the field intensity distribution in the region of the PJ with and without the TR clearly indicates that the focusing effect is caused by the presence of the TR^14–16^. A simplified model, considering that the TR acts as a focusing lens of the thickness and aperture corresponding to the TR dimensions, has been considered in supplementary materials. This model being too simplified for a quantitative comparison with the experiments and simulations, gives, however, good qualitative insight into the dependences of the focal length on incident angle and wavelength. This model captures essential features of these dependences observed in the experiments and simulations (see Fig. S2).

**Figure 3:**
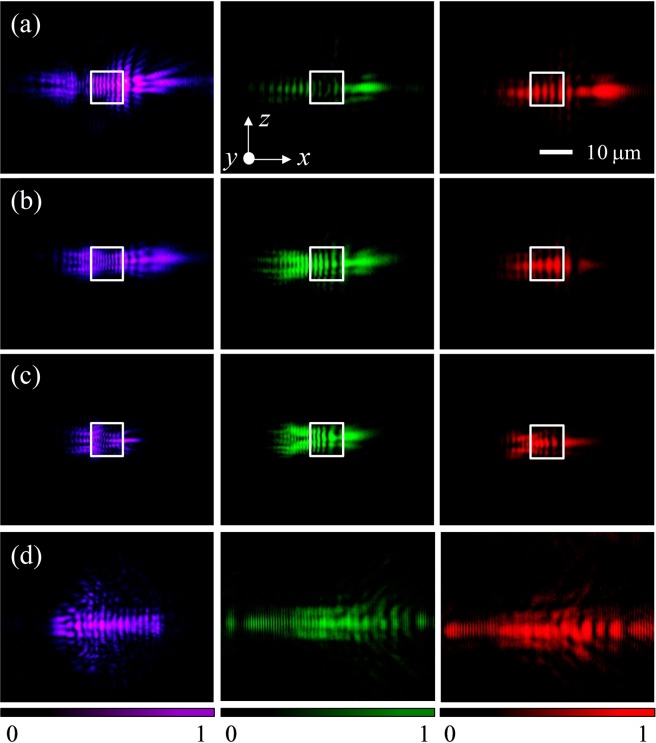
Raw experimental images of the flat dielectric thin rectangle in reflection mode at incident angles (**a**) 45°, (**b**) 60°, and (**c**) 75°. (**d**) Raw experimental images of the bare surface in the reflection at 45° incident angle. The incident wavelengths are 405 nm (left column), 532 nm (middle column), and 671 nm (right column).

In order to experimentally evaluate the focusing performance of the TR in reflection mode under different oblique incident angles, we have made appropriate measurements. Figure 3 shows the raw experimental images of the flat dielectric TR in reflection mode at incident angles of 45°, 60°, and 75° for incident wavelengths 405 nm, 532 nm, and 671 nm. All raw experimental images are captured on the *xz*-plane. The raw experimental images of the bare surface (without the TR) in the reflection at 45° incident angle are shown in Fig. 3(d) to demonstrate the focusing effect. In Fig. 3(c), it is clearly visible that the optical focusing of reflected beam appears on the right side of the TR. Comparison of Fig. 3(c,d) leads to conclude that the field localization is due to the presence of the dielectric TR. It should be noted that the effective size of the TR proportionally decreases with increasing wavelength of the incident radiation. This leads to a change in the focal length *f*_*x*_, as was previously predicted theoretically^14–16^.

**Figure 4:**
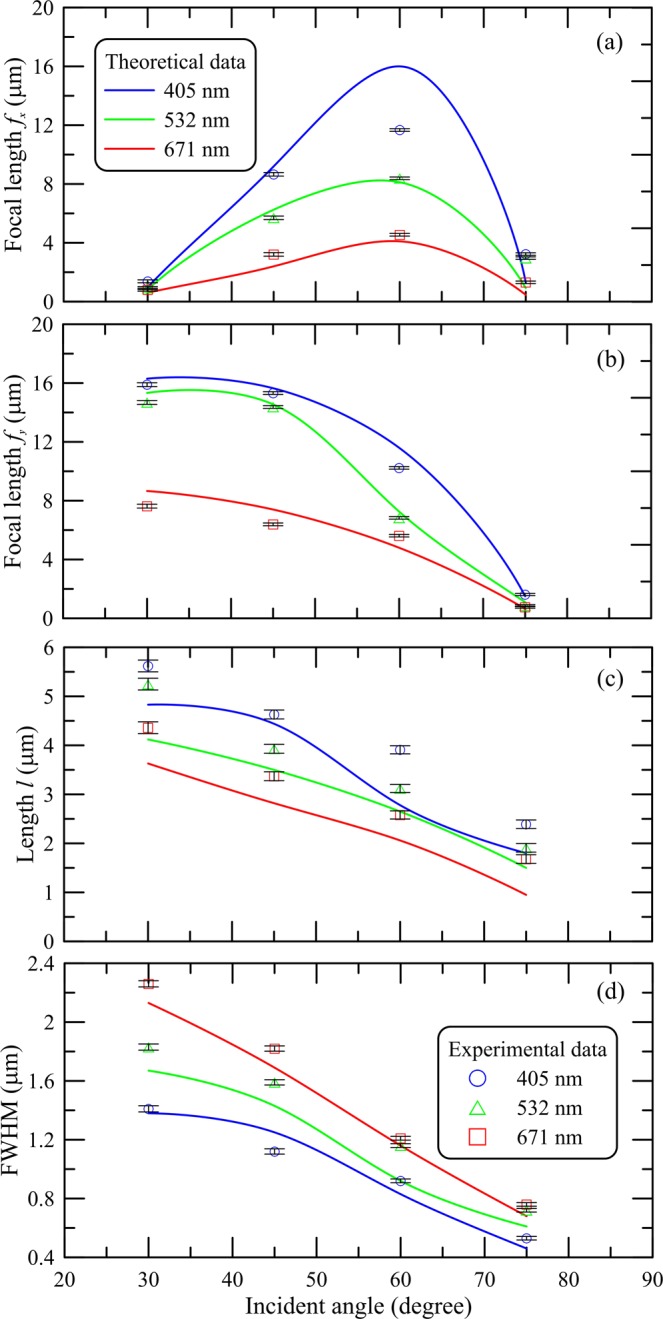
Focal lengths (**a**) *f*_*x*_, (**b**) *f*_*y*_, (**c**) Length, and (**d**) FWHM as a function of incident angle for the flat dielectric thin rectangle in reflection mode at different incident wavelengths.

Figure 4 shows the main key parameters as a function of incident angle for the flat dielectric TR in reflection mode at different incident wavelengths. The PJ length *l* is the radial distance from the point of maximum intensity at which the intensity distribution decays to 1/*e* of the point of maximum intensity. The FWHM is the double distance perpendicular to the propagation direction between the point of maximum intensity and half-maximum point. As expected, the minimal FWHM is observed at high angle (75°) of different illuminations. The FWHMs for illuminating wavelengths of 405 nm, 532 nm and 671 nm are 0.53 μm, 0.72 μm, and 0.76 μm, respectively. In Fig. 4(a), the local maximum near the incident angle of 60° can be explained by the proximity of incident angle to the Brewster angle θ_*b*_ for the selected TR material (*n* = 1.41, θ_*b*_ ~ 54.65°). From the presented experimental results, it is obvious that the position and geometric dimensions of PJ can be adjusted in space by changing the incident angle. The general tendency toward a decrease in the key parameters of the PJ with increasing incident wavelength is explained by a corresponding decrease in the longitudinal size of the TR, which follows from the previous results^22^.

## Conclusion

In conclusion, we have demonstrated a new concept of flat focusing mirror based on PJ effect in reflection mode at the first time. This type of near-field reflective focusing device can implement planar focusing structure without predefined optical axis. It has been shown that it is possible to control the position in space and dimensions of the PJ by simply changing the incident angle and wavelength. The structure has flat dielectric-metal configuration and is designed to function as a focusing flat mirror in the visible waveband. Using dielectric TR with size of *l* = 10 μm and thickness of *h* = 1 μm arranged on the top of the silicon wafer, we have realized a flat mirror to focus an incident beam with the focal length *f*_*x*_ changing from 1.38 μm to 11.67 μm when tuning the light incidence from 30° to 75°. The focusing properties of such a mirror are preserved in the wavelength range of 405 nm to 671 nm. As the focusing distances are in the micrometer range, it is possible to build small-sized photonic devices in which reflected beams could propagate diffraction-free at the distance of the PJ length without a waveguide structure. The condition of the PJ formation for the mesoscale dielectric structure must be met^14,22^, therefore, its minimum size is one wavelength of incident radiation. If it is desirable to obtain the PJ near the surface of the dielectric structure, several solutions may be used: (a) smaller dielectric dimensions close to a wavelength, (b) dielectric material with higher refractive index, and (c) the selection of the operational wavelength. The PJ in reflection mode demonstrates a long focal length which hardly achieved by a planar hyperlens^23^ or a flat lens^24^. The FFM presented here may be applied to enhance the radiation performance and novel optical imaging techniques.

## Methods

### Simulations

To simulate the relevance of the FFM concept, the performance of the TR in reflection mode has been numerically computed using rigorous calculations in the TE mode. The distribution fields of light propagations are solved using finite-difference time-domain (FDTD) method with perfectly matched layers as numerical boundary conditions^25^. The plane waves of 405 nm, 532 nm, and 671 nm wavelengths with unit intensity are incident onto the top surface of the TR under oblique angles. A mesh size of 20 nm is used in all simulations which is fine enough for computational accuracy. The calculations are performed by using the central processing unit of Intel Core i7 and random-access memory of 32 GB. The power flow patterns for the flat dielectric TR in reflection mode are generated by using Matlab program. All power flow patterns are normalized to incident power flow. The incident beam operated in reflection mode actually possesses scattering losses of optical power from the flat surface of the TR. In our simulations, this full-field scattering does not account from the intensity distribution. We are going to construct the three-dimensional scattering simulation. The full-field scattering from the flat surface can be completely understood.

### Sample fabrication

The fabrication of PDMS thin rectangle is based on lithography and replica molding process^26^. The micro-scale hole-type pattern is formed on a bare silicon wafer by using a mask aligner and exposure system. For casting PDMS thin rectangle, the hole-type pattern is operated as a pattern transfer agent. A liquid PDMS mixture is injected into the hole-type pattern and then another bare wafer is covered on it. The solidified PDMS thin rectangle is stripped from the hole-type pattern after baking at 85 °C for 20 minutes. Figure 1(a) shows the microphotograph of a single dielectric TR on a substrate. The height *h* and length *l* of the TR are approximately 1 μm and 10 μm, respectively.

### Characterization

The experimental configuration of scanning microscope system, designed for optical imaging, is shown in Fig. 1(b). The light sources are continuous-wave diode-pumped solid-state lasers with linear polarization (CNI Ltd. MDL-III-405, PGL-V-H-532, MRL-III-671). The laser beams of 405 nm, 532 nm and 671 nm wavelengths are illuminated the dielectric structure under oblique angles. The dividing plate is used to fasten laser head and adjust the incident angle of laser beam by steps of 15°. Due to obscuration of the incident light by the microscope mechanism, the smallest incident angle is 30°. The high-resolution digital camera is employed to capture the direct images of intensity maps from the dielectric structure. The section images of PJ along the *y* direction are implemented by a piezoelectric actuator with 10 nm step. A more detailed specification of the scanning microscope system can be found in previous literature^27^.

## Supplementary information

Supplementary information.

## Data Availability

All data generated or analysed during this study are included in this published article.
