# Supplementary material for: Experimental observation of flat focusing mirror based on photonic jet effect
Source: Sci Rep. 2020 May 21;10:8459. doi: 10.1038/s41598-020-65292-7 (PMC7242355; doi:10.1038/s41598-020-65292-7)
Supplement: Supplementary file 1 — Supplementary information. [file 41598_2020_65292_MOESM1_ESM.docx]

**Experimental observation of flat focusing mirror based on photonic jet effect**

Igor V. Minin^1,2^, Cheng-Yang Liu^3,*^, Yu-Chih Yang^3^, Kestutis Staliunas^4,5^ and Oleg V. Minin^1,2^

^1^Tomsk State Politechnical University, Tomsk, 36 Lenin Avenue, 634050, Russia.

^2^Tomsk State University, Tomsk, 30 Lenin Avenue, 634050, Russia.

^3^Department of Biomedical Engineering, National Yang-Ming University, Taipei City, 11221, Taiwan.

^4^ICREA, Passeig Lluís Companys 23, 08010, Barcelona, Spain.

^5^UPC, Dep. de Fisica, Rambla Sant Nebridi 22, 08222, Terrassa (Barcelona), Spain.

*Correspondence: cyliu66@ym.edu.tw (C.Y.L.)

**Supplementary materials**


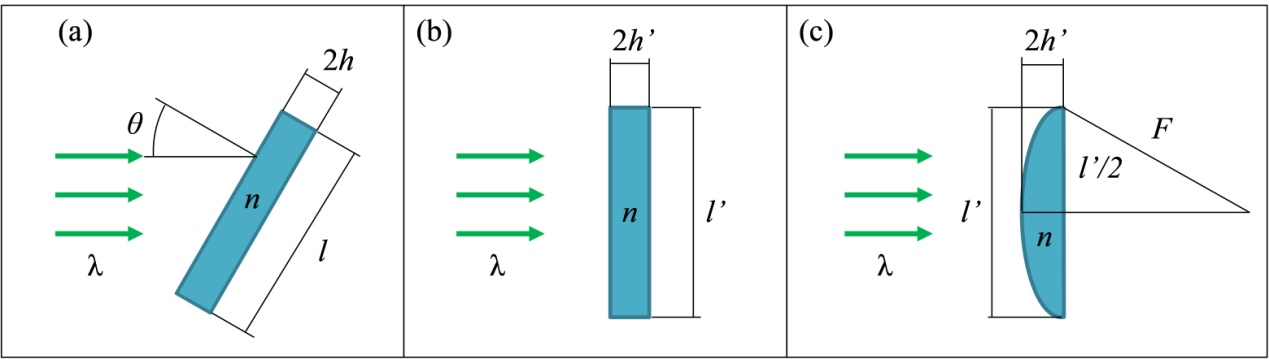


Figure S1. Geometrical models for simple analytic estimations of focusing dependence on incident angle.

In a simple geometrical optics approach, the oblique illumination of a flat plate (Fig. S1a) is equivalent to normal illumination of a plate with effective dimensions (Fig. S1b). The tilting of the plate is equivalent to the increase of its operational thickness *h*’ = *h/*cos*θ* and the decrease of its transverse dimensions *l*’= *l*cos*θ*. Simulating the plate by the convex-flat lens (Fig. S1c) leads to a rough estimation of the focal length:

${(F-2\left( n-1 \right)h')}^{2}+\frac{l^{'2}}{4}=F^{2}$ (S1)

and

$F=\left( \frac{l^{'2}}{4}+\left( 2{\left( n-1 \right)h}^{'} \right)^{2} \right)/4nh'$ (S2)

which results to explicit dependence of focal length on the incidence angle:

$F=\left( \left( \frac{\mathrm{lcos}\theta}{2} \right)^{2}+\left( \frac{2\left( n-1 \right)h}{\cos\theta} \right)^{2} \right)/(\frac{4\left( n-1 \right)h}{\cos\theta})$ (S3)

The *x* and *y* components of focal length follow from the equation (S3) as:

*f_x_* = *F*sin*θ*, *f_y_* = *F*cos*θ* (S4)

The dependences of focal lengths on the incident angles are plotted in Fig. S2. Note that the tendencies correspond well with the FDTD simulations and experimental results.


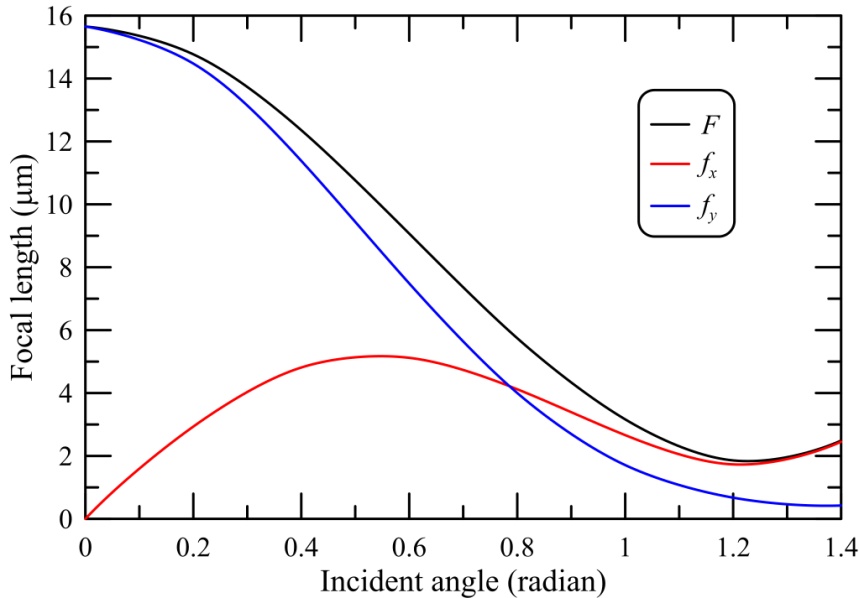


Figure S2. Focal lengths as a function of incident angle for analytic estimations.

Furthermore, the beam waist of the focus can be expressed as:

*w* = 1.2λ*F* / *l* (S5)

Figure S3 shows the beam waist as a function of incident angle for different incident wavelengths of the radiation. The tendency is that the beam waist decreases as the incident angle increases. This analytic estimation is also in a good agreement with the FDTD simulations and experimental data.


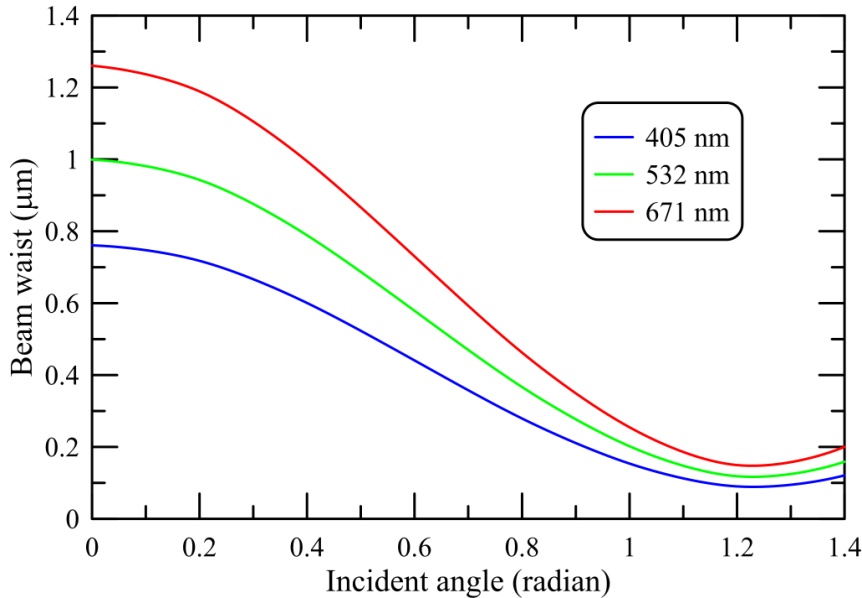


Figure S3. Beam waist as a function of incident angle for analytic estimations.
